# Emerging role of *KDM5C* in X-linked intellectual disability based on human genetic data and zebrafish models

**DOI:** 10.3389/fnmol.2026.1750311

**Published:** 2026-02-10

**Authors:** Baoqiong Liao, Meihuan Chen, Yun Huang, Mei Shuai, Liangpu Xu, Shuwen He, Hailong Huang

**Affiliations:** 1 College of Clinical Medicine for Obstetrics & Gynecology and Pediatrics, Fujian Medical University, Fuzhou, Fujian, China; 2 Ganzhou Maternal and Child Health Hospital, Ganzhou, Jiangxi, China; 3 Fujian Provincial Key Laboratory of Prenatal Diagnosis and Birth Defects, Medical Genetic Diagnosis and Therapy Center of Fujian Maternity and Child Health Hospital, Fuzhou, China; 4 Department of Physiology, Development, and Neuroscience, University of Cambridge, Cambridge, United Kingdom; 5 Department of Chemistry and Molecular Biology, Faculty of Science, Gothenburg University, Gothenburg, Sweden

**Keywords:** Claes-Jensen syndrome, *KDM5C*, neuroimmunity, neuroinflammation, novel variants, X-linked intellectual disability

## Abstract

**Introduction:**

Claes-Jensen syndrome is a rare X-linked syndromic neurodevelopmental disorder by pathogenic variants in lysine specific demethylase 5C (*KDM5C*), a lysine-specific histone demethylase.

**Methods:**

In this study, clinical evaluations were conducted in affected individuals and carrier females. X-chromosome inactivation (XCI) assays were performed to assess genotype—phenotype correlations. Functional studies evaluated variant effects on RNA transcription, protein expression, and stability. Zebrafish models were used for *in vivo* validation. RNA sequencing with KEGG and GO analyses identified dysregulated genes and pathways, further confirmed in zebrafish.

**Results:**

Two novel *KDM5C* variants NM_004187.5:c.3019del and NM_004187.5:c.782-2A>T were identified in unrelated families with X-linked ID. Affected males presented with short stature, microcephaly, language delay, and intellectual disability, while carrier females showed milder features including learning difficulties and short stature. Skewed XCI in some carriers suggested a role in phenotypic variability. Both variants impair RNA transcription, protein expression and stability. Zebrafish models recapitulated neurodevelopmental and behavioral abnormalities. Transcriptomic analyses revealed disrupted antiviral and interferon-related signaling, implicating aberrant immune activation. Pharmacologic inhibition of the Toll-like receptor pathway ameliorated mutant phenotypes, highlighting neuroinflammation as a potential therapeutic target for *KDM5C*-related disorders.

**Conclusion:**

These findings expand the mutational spectrum of *KDM5C*-associated ID and uncover a novel pathogenic mechanism between *KDM5C* dysfunction, protein instability, and dysregulated inflammatory signaling.

## Introduction

Intellectual disability (ID) is characterized by significant impairments in both intellectual and adaptive functioning, including disorders in reasoning, problem solving, planning, abstract thinking, judgment, academic learning, and the capacity for personal independence and social responsibility developing (de Ligt et al., 2012). ID represents a major public health concern and affects approximately 1.5%-3% of the population worldwide (de Ligt et al., 2012). Although the human X chromosome carries only about 4% of the protein-coding genes in the human genome, X-linked gene variants are estimated to cause 8-12% of ID in males (Ropers and Hamel, 2005). Most heterozygous female carriers are asymptomatic, However some may exhibit mild learning difficulties due to X-chromosome inactivation (XCI) escape (Carmignac et al., 2020). To date, 200 genes have been associated with X-linked intellectual disability (XLID), according to the OMIM database (https://omim.org/accessed on 1 August 2025), with functions including transcription regulation, signal transduction, metabolism, components of membrane-associated roles, cytoskeleton, RNA processing, DNA metabolism, protein synthesis, ubiquitination, cell cycle and cell adhesion.

Claes-Jensen syndrome (OMIM 300534) is an X-linked syndromic neurodevelopmental disorders (NDDs) caused by pathogenic variants in the lysine specific demethylase 5C (*KDM5C*) gene (OMIM 314690). Inherited maternal and more rarely *de novo* variants explain about 0.7-2.8% of X-linked intellectual disability (Hatch et al., 2021). The *KDM5C* gene, also known as JARID1C or SMCX, encodes a specific H3K4me3 and H3K4me2 demethylase, functioning as a transcriptional repressor through the RE-1-silencing transcription factor (REST) complex (Tahiliani et al., 2007). The association between *KDM5C* mutations and disease was first reported in 2005 by Jensen et al. who identified seven variants in 20 affected males from seven families through mutational screen of brain expressed genes on the X chromosome in a cohort of families with X-linked ID (Jensen et al., 2005).

Located at Xp11.22, *KDM5C* contains 26 exons and encodes a transcription factor with several DNA binding motifs and histone demethylation activity specific for demethylated and trimethylated lysine 4 of histone H3 (H3K4). Previous studies suggest that *KDM5C* plays a central role in transcriptional repression and chromatin regulation during cell growth, differentiation, and development. In mouse models, loss of *KDM5C* result in cognitive impairment, dendritic spine morphological abnormalities, and widespread transcriptional dysregulation of neurodevelopmental genes. The female knockout mouse exhibit milder cognitive phenotypes characterized by memory deficits and learning disabilities (Scandaglia et al., 2017). Clinical manifestations reported in the OMIM database (accessed on 1 August 2025) include short stature, small forehead, prognathism, micrognathia, maxillary hypoplasia, facial hypotonia, flat philtrum, thin upper lip, high narrow palate, small and deep-set eyes, and large ears. Some affected individuals have also been reported to present with visual impairments, including strabismus, hypermetropia, and myopia, as well as skeletal anomalies involving the hands and feet.

*KDM5C* is known for its enzymatic demethylase activity, mediated by the JmjN and JmjC domains, which removes the activating histone marks H3K4me2/3 at gene promoters to repress transcription (Ram et al., 2011; Vallianatos et al., 2020; Hatch and Secombe, 2022). However, emerging evidence suggests that *KDM5C* may affect normal neuronal development and transcriptional programs through non-enzymatic mechanisms. Notably, not all *KDM5C* variations result in a reduction in histone demethylase activity (Vallianatos et al., 2018). Pathogenic missense mutations in KDM5C often spare its catalytic JmjC domain, instead localizing to regions essential for macromolecular assembly. These variants maintain intrinsic demethylase activity while ablating critical protein-protein interactions, implicating a pathogenesis rooted in disrupted complex formation rather than mere loss of enzymatic function (Peng et al., 2015). The precise molecular mechanism, however, remain unresolved. No single classical pathway fully explains Claes-Jensen syndrome. Instead, *KDM5C* sits upstream of multiple neuronal signaling programs by tuning chromatin states at their gene targets (Vallianatos et al., 2018; Hatch and Secombe, 2022).

As a recognized model for studying human neurodevelopmental disorders, zebrafish can effectively simulate phenotypes such as epilepsy, NDDs, and synaptic dysfunction (Vaz et al., 2019), which are highly correlated with Claes Jensen syndrome associated with *KDM5C*, highlighting the potential value of using zebrafish to study this disease. However, current research on the function of *KDM5C* in zebrafish is still very limited. So far, the most groundbreaking work only appeared in a paper published in the journal Cell in 2007 (Iwase et al., 2007), which for the first time revealed the role of *KDM5C* in neuronal survival and dendritic development, as well as its association with demethylase activity, in a zebrafish model. In the present study, we identified two families with ID, in which the probands exhibited severe intellectual disability. Whole-exome sequencing revealed novel *KDM5C* variants not previously reported in public databases. We constructed a *KDM5C-*zebrafish model to investigate the key role of the *KDM5C* gene in regulating neural development and behavior, and to explore the related signaling pathways of *KDM5C* affecting neural development, neuroinflammation and neuroimmune at the transcriptome level. By expanding the mutational spectrum of *KDM5C* and elucidating its contribution to Claes–Jensen syndrome, our findings provide new insights into disease pathogenesis and establish a foundation for future work toward mechanism-based therapeutic strategies.

## Materials and methods

### Ethical compliance

Informed consent was obtained from all patients or their legal guardians prior to enrollment in this study. All procedures were conducted in accordance with the ethical standards of the institutional and national research committees, as well as with the Declaration of Helsinki. The study protocol was approved by the Ethics Committee of Ganzhou Maternal and Child Health Hospital (Approval No. 2025-126, September 25, 2025).

### X-chromosome inactivation studies

XCI status was evaluated using the AR (CAG)n repeat assay. Fluorescently labeled PCR was performed on both intact genomic DNA and DNA digested with the methylation-sensitive restriction enzyme HpaII, following established methods (Bolduc et al., 2008). Because the inactive X chromosome is methylated at the HpaII restriction site, digestion prevents amplification of the active allele, allowing quantitative assessment of skewing. The XCI ratio was calculated from the relative peak heights of PCR products. XCI was considered random at ~50:50 (Harper, 2011), skewed at ≥80:20 and extremely skewed at ≥90:10 (Fieremans et al., 2016; Giorgio et al., 2017). Inactivation of the *KDM5C* gene was specifically assessed by PCR amplification of exon 1 from intact and HpaII-digested DNA.

### DNA isolation, whole-exome sequencing, and variant analysis

Peripheral blood samples were collected from the probands and their patents. Genomic DNA was extracted using the QIAamp DNA Mini Kit for blood (Qiagen, Hilden, Germany), following the method as previously described (Liao et al., 2025a,b; Xie et al., 2025). Trio-based WES was performed by KingMed Diagnostics (Guangzhou, China), and the platform is Illumina NextSeq 550, including exome library preparation, high-throughput sequencing, and data analysis. Variant were filtered based on clinical phenotypes of the affected subjects, allele frequencies in population databases (dbSNP, 1,000 Genome, ExAC), disease database (OMIM, HGMD, Clinvar), and predictive tools (SIFT, Polyphen2, Mutation Taster and Splice AI). Two novel *KDM5C* variants were identified: a frameshift variant (NM_004187.5: c.3019del) in Family 1 and a splice-site variant (NM_004187.5: c.782-2A>T) in Family 2. Both variants were validated by Sanger sequencing in available family members.

### Minigene splicing assay

A genomic fragment (exons 6-8) containing c.782-2A>T was amplified from the proband (Family 2, individual III-4) and then cloned into the pcDNA3.1(+) expression vector. The construct was transfected into HEK293 and HeLa cells. After 48 h, total RNA was extracted with TRIzol (TaKaRa, Japan). Splicing outcomes were subsequently analyzed via RT-PCR and Sanger sequencing.

### Protein structure prediction

The amino acid sequence of *KDM5C* (1562 residues) was downloaded from UniProt web (https://www.uniprot.org/). Three-dimensional protein structures of wild-type and mutant proteins (c.3019del and c.782-2A>T) were predicted using AlphaFold 3 web server (https://golgi.sandbox.google.com/about) (Lomize et al., 2022). The best model was selected based on predicted local distance difference test (pLDDT) scores. Protein models were visualized and refined using PyMOL (https://pymol.org/).

### *In vitro* functional studies

Full-length *KDM5C* cDNA (WT, c.3019del, and c.782-2A>T) were cloned into pc.DNA3.1 (+) with an N-terminal FLAG tag. HEK293 cells were cultured in 90% MEM supplemented with 10% FBS and penicillin-streptomycin at 37 °C, 5% CO_2_, and transfected using Opti-MEM (Lonza, Bassel, Switzerland) and Lipofectamine 3000 (Carlsbad, CA, United States). After transfection for 48 h, the samples were collected for quantitative polymerase chain reaction (qPCR) and western blot (WB) detection, respectively.

### qPCR, WB, and protein stability assay

RNAs were isolated from HEK293 cells of each group using Trizol reagent according to the manufacturer,s directions. Take 1μg RNA and use NovoScript^®^ Plus All-in-one 1st Strand cDNA Synthesis SuperMix (Novoprotein, China) Synthesize cDNA. qPCR detection was performed using the ArchimedTM platform (Kunpeng Gene Technology Co., Ltd., China), and ChamQ Universal SYBR qPCR Master Mix (Vazyme, China) was used for qPCR analysis of the mRNA expression of *KDM5C* gene (primers are shown in Supplementary Table 1). The results of qPCR were calculated using relative quantification method, and the gene expression level F = 2^−ΔΔ^CT. The total protein was extracted from HEK293 cells of each group, add an appropriate amount of RIPA lysis buffer (Beyotime, China) to extract total protein, add 5 × SDS loading buffer (Sangon, China) to denature at 100 °C, perform SDS-PAGE electrophoresis, transfer to NC membrane (Merck, Germany), block with 5% BSA (Solarbio, China) for 2 hours, and use primary antibody Flag (1:5000, Proteintech, China), H3K4me2/3 (1:2000, Abcam, USA). The internal reference GAPDH (1:5000, Proteintech, China) and Histon 3 (Abcam, USA) were incubated overnight at 4 °C, and the secondary antibody (1:10000, Proteintech, China) was incubated at 37 °C for 2 h, washed 3 times, and photographed using an Enhanced Chemiluminescence chemiluminescence spectrophotometer (Servicebio, China). The optical density values of the target protein and internal reference were analyzed using Image J. After transfection of each group of cells for 48 hours, protein stability was assessed by 20 μg/mL cycloheximide (Aladdin, China) chase for 0, 1, 2, 4, 8, or 24 h, followed by WB analysis.

Total RNA was extracted using RNAiso Plus reagent (Takara, China) and reverse-transcribed with a NovoScript^®^ first Strand cDNA Synthesis SuperMix kit (Novoprotein, China). Gene expression was assessed by quantitative PCR. For protein analysis, histones were extracted, and western blotting was performed to detect FLAG-tagged *KDM5C* and H3K4me2/3 with GAPDH as the loading control. Proteins were separated by SDS-PAGE, transferred to PVDF membranes, and incubated overnight at 4 °C with primary antibodies (FLAG, SanYing 80801-2-RR; H3K4me3, Abcam ab8580; H3K4me2, Abcam ab32356), followed by 1 h incubation with secondary antibodies at 37 °C. Signals were visualized via chemiluminescence and quantified semi-quantitatively. Protein stability was assessed by cycloheximide (20 μg/mL) chase for 0, 1, 2, 4, 8, or 24 h, followed by Western blotting analysis.

### Transcriptomic assay, differential expression assay and functional enrichment analysis

Collect cells from each group after 48 hours of transfection, and conduct three repeated experiments for each group. The RNA was extracted with Trizol reagent (Takara, Japan), and its purity and integrity were detected with NanoPhotometer spectrophotometer and agarose gel electrophoresis. Subsequently, Illumina's NEBNext^®^ UltraTM RNA Library Construction Kit (NEB, E3330S) was used to construct an RNA library, which was sequenced on the Illumina platform to obtain a paired end sequence of 150 bp in length. These paired end sequences were aligned and analyzed with the reference genome using the HISAT2 program. Using DESeq2 software, genes with *P* < 0.05 and | Log2FC |≥3 were identified as differentially expressed genes. Further use clusterProfiler (3.8.1) software to perform GO functional enrichment analysis and KEGG pathway enrichment analysis on these differentially expressed genes. GO functional enrichment primarily encompasses biological processes (BP), cellular component (CC), and molecular function (MF).

RNAs were isolated from HEK293 cells of each group. Sequencing libraries were generated using Hieff NGS Ultima Dual-mode mRNA Library Prep Kit for Illumina (Yeasen Biotechnology Shanghai, China), and sequenced on an Illumina NovaSeq platform to generate 150 bp paired-end reads. GO functional enrichment and KEGG pathway enrichment analysis of differentially expressed genes were conducted using the cluster Profiler (3.8.1) software. GO terms with corrected *P* values less than 0.05 were considered significantly enriched by differentially expressed genes. GO functional enrichment primarily encompasses biological processes (BP), cellular component (CC), and molecular function (MF).

### *In vivo* functional studies in zebrafish

The wild-type zebrafish strain (AB) was obtained from National Zebrafish Resource Center and maintained under standard laboratory conditions. Zebrafish Embryos and larvae were cultured in 10-cm plastic petri dishes in an incubator maintained at 28.5 °C. Embryos were raised in E3 medium, containing (in mM) 5.0 NaCl, 0.17 KCl, 0.33 MgSO_4_, 0.33 CaCl_2_. All procedures involving experimental animals were conducted in strict accordance with institutional and national guidelines and regulations for the care and use of laboratory animals.

### Microinjection, human *KDM5C* variants over-expression and rescue experiment in zebrafish

The *KDM5C* gene sequence (GenBank: NM_004187.5) was obtained from NCBI, and the CDS region of the *KDM5C* gene and its mutation sequences c.3019del and c.782-2A>T were subsequently inserted into the pcDNA3.1 (+) plasmid. After transcribing this plasmid into RNA using the T7 promoter transcription kit, these RNAs were injected into the fertilized egg at different doses to determine the optimal injection dose. The experimental results showed that when the injection doses of *KDM5C* mRNA or *KDM5C* c.782-2A>T or *KDM5C* c.3019del mRNA at 100 ng/μL, 300 ng/μL, or 600 ng/μL, there were differences in the mortality and malformation rate exhibited by the embryos (Exploring the optimal concentration are shown in Supplementary Table 2). 300 ng/μL of *KDM5C* mRNA or *KDM5C* c.782-2A>T or *KDM5C* c.3019del mRNA was used for each injection in zebrafish experiments,.

One-cell stage zebrafish embryos were injected with 300 ng/μL of mRNAs: Control (RFP), *KDM5C* mRNA(WT), *KDM5C* c.782-2A>T, *KDM5C* c.3019del or WT plus respective mutant for rescue. Embryos were cultured in E3 at 28.5 °C to 48 h post fertilization (hpf). Total RNA from 10 embryos per group was extracted, reverse-transcribed, and analyzed by qPCR (primers are shown in Supplementary Table 3).

### Toll-like signaling pathway inhibitor (CU-CPT 4a) treatment

As a Toll-like signaling pathway inhibitor, CU-CPT 4a was selected for intervention.

One-cell stage zebrafish embryos were microinjected with WT or mutant *KDM5C* mRNAs and cultured at 28.5 °C. CU-CPT 4a (½ LC50) was administered 24 h post-injection. Eight groups were established: Control, Control+CU-CPT 4a, WT, WT+CU-CPT 4a, c.3019del, c.3019del+CU-CPT 4a, c.782-2A>T, and c.782-2A>T+CU-CPT 4a. At 24 hpf, embryos were collected for RNA extraction and qPCR analysis of six TLR pathway genes (TLR3, NFKB1, IFNB1, IRF7, SAT1a, SAT1b).

### qPCR analysis of overexpression effect

The zebrafish of qPCR experimental method is the same as the cell experiment section. When the zebrafish developed to 24 h post fertilization (hpf), 10 embryos were taken from each group for RNA extraction, reverse transcription, and qPCR experiment (primers are shown in Supplementary Table 4).

### White light phenotype analysis and alcian blue staining

Soak 72 hpf zebrafish embryos in a 0.03% MS-222 solution (3-aminobenzoate ethyl ester; Sigma, USA) for 5 minutes to induce anesthesia. Then, the SMZ800N stereomicroscope (Nikon, Japan) was used to take photos of the ventral side at 3 times magnification and the dorsal side at 6 times magnification to observe their developmental characteristics such as body length, eye and head area. Image J software was used to calculate body length, eye head area (*n* = 10 per group). 120 hpf zebrafish were fixed in 4% paraformaldehyde at 4 °C overnight, bleached, and treated with acidic alcohol. Samples were stained in 0.05% Alcian Blue 8GX (Solarbio, China) in 5% acetic acid for 16-24 h at room temperature. Post-staining, larvae were rehydrated through a graded ethanol series (75%, 50%, 25%) and 1 × PBST (30 min each), then digested in 2% boric acid with 1% trypsin at 32 °C until tissues were transparent and cartilage clearly visible. Samples were further cleared in 25%, 50%, and 75% glycerol in 0.5% KOH overnight and stored in 100% glycerol at 4 °C. Place the samples under an SMZ800N stereomicroscope (Nikon, Japan) at a magnification of 6 times and analyze the stained area using Image J software (*n* = 10 per group).

### Behavioral assays in zebrafish larvae

Zebrafish larvae were incubated until 96 hpf and then transferred individually into 96-well plates for culture until 120 hpf. Fifteen larvae per group were monitored for 5 min to assess locomotor behavior. Behavioral trajectories were recorded using the DanioVision Video-Track system (Noldus, Germany) and analyzed with EthoVision XT 17 (Noldus, Germany) to quantify movement patterns, swimming distances, and average speeds.

### Statistical analysis

All data are derived from three independent experiments and are expressed as mean ± SD. Data analysis was performed using GraphPad Prism 10.0. One-way analysis of variance (ANOVA) was used to compare data among groups. The basic principle of choosing analysis of variance is that this test is applicable to comparing whether there is a significant difference in the overall mean of three or more independent experimental groups. Tukey,s multiple-comparison test was used to test the difference between groups subsequently. A *p* value of less than 0.05 was considered statistically significant.

## Results

### Clinical presentation

**Figure 1:**
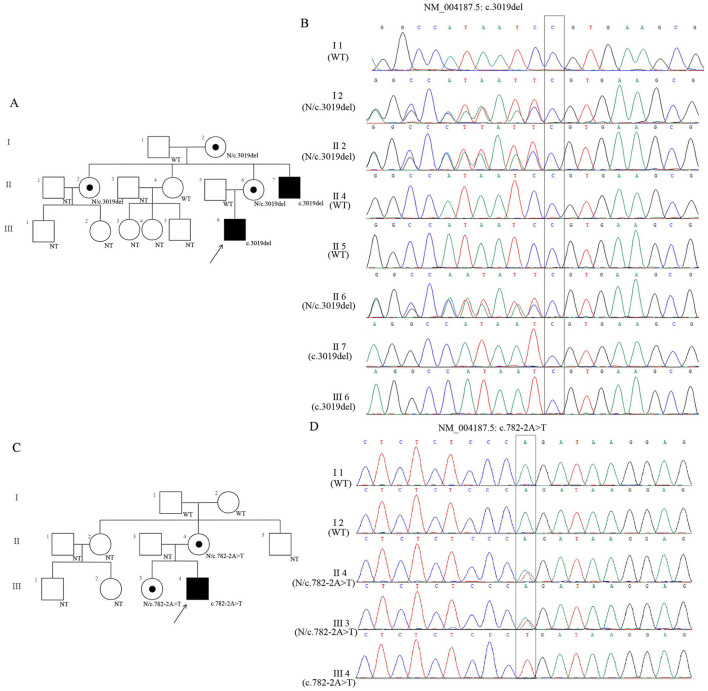
Genetic analysis of two families with *KDM5C* variants. **(A)** Pedigree of Family 1 carrying the *KDM5C* variant (c.3019del). Circles and squares represent females and males, respectively. The proband is indicated by an arrow. Fully shaded symbols denote affected individuals with ID, while half-shaded symbols indicate heterozygous carriers. “WT” and “N” indicate *KDM5C* gene without the variant. NT, is for “not available to be tested”. **(B)** Sanger sequencing chromatograms of Family 1 showing the c.3019del variant. The black box highlights the affected nucleotide position. **(C)** Pedigree of Family 2 carrying the *KDM5C* variant (c.782-2A>T), annotated as in **(A)**. **(D)** Sanger sequencing chromatograms of Family 2 showing the c.782-2A>T variant, with the black box marking the altered nucleotide.

This study investigated two unrelated Chinese families presenting with neurodevelopmental disorders (Figure 1). Proband 1 (Family 1, III-6, c.3019del), a 5 year 7 month old male, is the only child of non-consanguineous and healthy parents. He walked at 12 months and spoke first words at 3 years old, presenting with severe expressive language delay and ID. Speech is currently limited to simple repetitive words. His parents were clinically normal. A maternal uncle (II-2, N/3019del) presented with severe ID and short stature (148 cm, <3rd percentile), and a maternal aunt (II-7, c.3019del) had mild ID and short stature (150 cm, <3rd percentile). Physical examination of the proband revealed height 105 cm (−2 SD), weight 15 kg (−2 SD), and occipitofrontal circumference 48 cm (−3 SD). He was frequently excited and displayed a persistent smiling expression (Figures 1A, B).

Proband 2 (Family 2, III-4, c.782-2A>T), a 5 year 11 month old male, walked at 18 months and spoke at 2 years old. He was diagnosed with severe ID, with speech limited to a few isolated words. His mother (II-4, N/c.782-2A>T) exhibited learning difficulties, impaired mathematical skills, and short stature (143 cm, **<** 3rd percentile) whereas his older sister (III-3, N/c.782-2A>T), 8 years 5 months, was short (107 cm, <3^rd^ percentile) but safe. Physical examination of Proband 2 showed height 101 cm (−3 SD), weight 15 kg (−3 SD), and an occipitofrontal circumference of 49 cm (−2 SD). His grandparents showed normal intelligence and stature (I-1/I-2, WT). The child was noted to be calm and cheerful (Figures 1C, D).

### XCI assay

XCI patterns was evaluated using the androgen receptor (AR) methylation assay to investigate potential skewing associated with the *KDM5C* variants. In Family 1, the X chromosome carrying the c.3019del mutation, inherited from the mother, was completely inactivated (100%) in the proband's mother (II-6). In addition, preferential skewing of XCI was observed in other female carriers within the family: the proband's aunt (II-2) and grandmother (I-2) exhibited 85% skewing of the mutant allele, indicating a non-random inactivation pattern (Supplementary Figure 1A). In Family 2, the X chromosome harboring the c.782-2A>T mutation, also maternally inherited, displayed complete skewing (100%) in both the proband's mother (II-4) and sister (III-3) (Supplementary Figure 1B). These results suggest that female carriers of these *KDM5C* variants preferentially inactivate the X chromosome carrying the mutant allele, which may contribute to variable phenotypic expression and reduced penetrance in heterozygous females.

### Minigene splicing assay

*KDM5C* is predominantly expressed in the nervous system. Due to ethical constraints, only peripheral blood samples could be obtained from the affected individuals. Given the low expression levels of *KDM5C* in peripheral blood, the minigene assay was performed to investigate the functional consequence of the NM_004187.5: c.782-2A>T variant on pre-mRNA splicing. Subsequently, RT-PCR results revealed an aberrant splice product characterized by the deletion of five nucleotides (AAGAT) at the exon 6-exon 7 junction (Supplementary Figure 2A). This splicing alteration was not detected in transcripts derived from control constructs. The expression of the 5bp (AAGAT) deletion between Exon6 and Exon7 at the cDNA levels is as follows: NM_004187.5(*KDM5C*):c.1097_1101del, which is predicted to cause a frameshift and induce a premature termination codon (PTC) within exon 7, and protein levels is designated as p.(Trp366Leufs^*^10) (Supplementary Figures 3B, C). This variant is expected to result in a truncated protein consisting of 375 amino acids, potentially resulting in loss of function.

### Effect of mutations on protein structure, RNA expression, protein expression, protein stability

To evaluate the structural impact of the two novel *KDM5C* variants NM_004187.5: c.3019del and NM_004187.5: c.782-2A>T, the predicted protein models were generated using the AlphaFold web server (https://golgi.sandbox.google.com/about). The NM_004187.5: c.3019del variant is predicted to cause a frameshift, resulting in a truncated *KDM5C* protein comprising 1,054 of the 1,561 amino acids of the mature protein. This frameshift mutation is likely to significantly alter the three-dimensional protein structure of *KDM5C* (Supplementary Figure 3A). Similarly, The NM_004187.5: c.782-2A>T variant will produce a truncated protein consisting of only 357 of the 1561 amino acids, corresponding to a significant loss of the C-terminal domains (Supplementary Figure 3B). These structural alterations would disrupt the native folding of *KDM5C* protein, compromising the protein stability and impairing its binding capacity. The impact of *KDM5C* variants on RNA and protein levels was assessed in HEK293 cells transfected with WT or mutant constructs qRT-PCR revealed reduced *KDM5C* mRNA in both c.3019del and c.782-2A>T compared with WT (Supplementary Figures 3D–E). At the protein level, c.3019del was markedly decreased, whereas c.782-2A>T remained comparable to WT (Supplementary Figure 3F).

### Effect of mutations on H3K4me2/3 expression and protein stability

Histone demethylase activity was indirectly assessed by quantifying H3K4me2/3 relative to total H3 and H4, showing no significant changes for either variant, indicating retained catalytic activity (Supplementary Figures 4A–C). Protein stability was evaluated using cycloheximide (0–24 h). At 24 h, WT levels decreased to ~75%, whereas c.3019del and c.782-2A>T levels decreased to ~30%. Notably, c.782-2A>T protein remained stable for the first 8 h, similar to WT (Supplementary Figures 4D, E). These results indicate that both variants substantially compromise *KDM5C* protein stability.

### *In vivo* analysis of *KDM5C* variants in Zebrafish model

**Figure 2:**
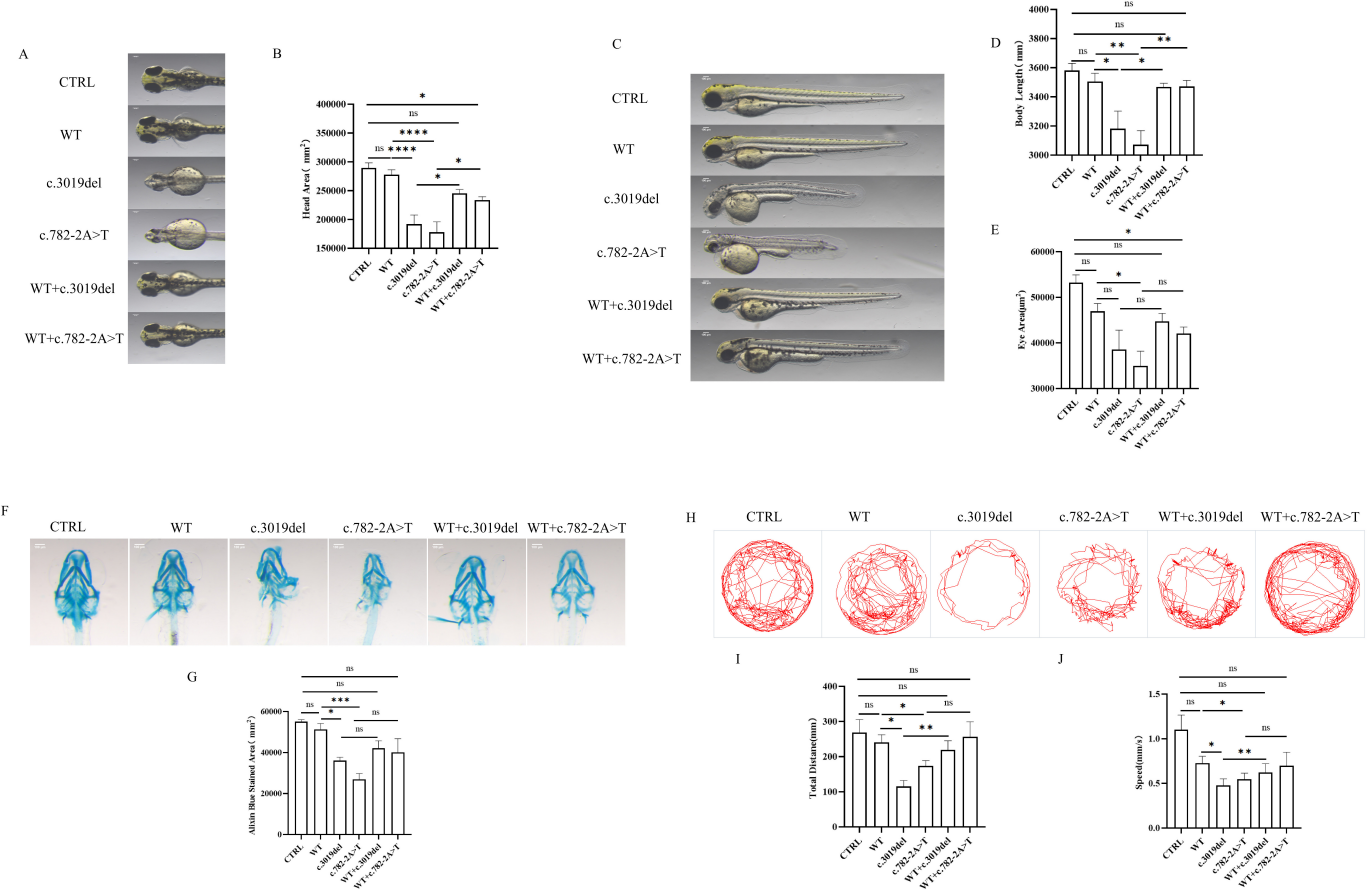
Phenotypic consequences of *KDM5C* variants and behavioral assays of zebrafish larvae in zebrafish models. **(A, C)** Representative phenotypic images of six groups at 72 hpf. Embryos expressing *KDM5C* c.3019del or *KDM5C* c.782-2A>T exhibited reduced body length, head size, and eye area compared with control and wild-type (WT) groups. Co-injection with WT *KDM5C* mRNA rescued these phenotypic defects. Scale bar = 500 μm. **(B)** Quantification of head area (*n* =10 per group). **(D)** Quantification of body length (*n* =10 per group). **(E)** Quantification of eye area (*n* =10 per group). **(F)** Representative Alcian blue staining images of six groups at 120 hpf. Both *KDM5C* variants significantly reduced cartilage size and disrupted cartilage morphology. Scale bar = 500 μm. **(G)** Quantification of Alcian blue–stained cartilage area (*n* =3 per group). Data are expressed as mean ± SD. no significant difference; ^*^p < 0.05; ^**^p < 0.01; ^***^p < 0.001. **(H)** Representative locomotor tracking plots for six experimental groups at 120 hpf. **(I)** Quantification of total distance traveled by larvae (*n* =10 per group) over time. **(J)** Quantification of swimming speed for larvae (*n* =10 per group). ANOVA was used to compare data among groups. Data are expressed as mean ± SD. ns, no significant, ^*^p < 0.05; ^**^p < 0.01; ^***^p < 0.001; ^****^P < 0.0001.

To assess the effects of *KDM5C* variants *in vivo*, overexpression and rescue models of c.3019del and c.782-2A>T were generated in zebrafish model. Both variants significantly reduced head area, body length, and eye size compared with control and WT groups (Figures 2A–E). These morphological deficits were effectively rescued by co-injection of WT *KDM5C*. Alcian Blue staining revealed impaired cartilage development in c.3019del and c.782-2A>T larvae, which were largely restored in rescue experiments (Figures 2F, G), indicating that role of *KDM5C* in craniofacial and cartilage formation.

Behavior in zebrafish is a sensitive indicator of neurodevelopment. The representative tracks suggest that *KDM5C* variants affect the swimming behavior in zebrafish (Figure 2H). All behavioral parameters were significantly altered in the c.3019del and c.782-2A>T groups compared with the WT group, including total distance traveled and swimming speed (Figures 2I, J). After rescue with WT mRNA injection, the c.782-2A>T+WT and c.3019del+WT groups both showed improved locomotor behavior. Notably, the c.3019del+WT group showed a significant improvement compared with the c.3019del group, indicating that WT mRNA partially rescued the behavioral deficits in c.3019del zebrafish. However, there was no significant difference between c.782-2A>T and c.782-2A>T+WT, suggesting that WT mRNA injection did not further alter behavior in the c.782-2A>T group.

Histone demethylase activity, assessed via H3K4me3 and H3K4me2 levels relative to total histone H3/H4 was unchanged across groups, consistent with *in vitro* results (Supplementary Figures 5A–C). It indicate that the variants impair neurodevelopment through mechanisms independent of catalytic activity.

### GO pathway and KEGG pathway analysis

To assess *KDM5C*'s role in neural development, differentially expressed genes (DEGs) were analyzed in the HEK293 cells expressing c.3019del or c.782-2A>T variants vs. controls. c.3019del cells exhibited 363 DEGs (355 upregulated, 8 downregulated), whereas the c.782-2A>T group had 326 DEGs (320 upregulated, 6 downregulated) (Supplementary Figures 6A, B, E, F).

GO analysis revealed that both variants were enriched in biological processes related to antiviral and interferon responses, including defense response to virus, response to virus, response to type I interferon, interferon-mediated signaling pathway, and cellular response to type I interferon. Key molecular functions included cytokine activity, type I interferon receptor binding, cytokine receptor binding, double-stranded RNA binding, and RNA helicase activity (Supplementary Figures 6C, G.)

KEGG pathway analysis showed that c.3019del DEGs were mainly associated with Influenza A, RIG-I-like receptor signaling, Coronavirus disease–COVID-19, Hepatitis C, NOD-like receptor signaling, and Toll-like receptor signaling. c.782-2A>T DEGs were enriched in RIG-I-like receptor signaling, Influenza A, NOD-like receptor signaling, Herpes simplex virus 1 infection, Coronavirus disease–COVID-19, and Cytosolic DNA-sensing pathway (Supplementary Figures 6D, H). These results suggest that both *KDM5C* variants prominently affect antiviral and interferon-related pathways, implicating immune signaling in *KDM5C*-mediated neural development.

### Improvement effects of TLR signaling pathway inhibitor (CU-CPT 4a) on abnormal phenotypes and behaviors induced by *KDM5C* variants

**Figure 3:**
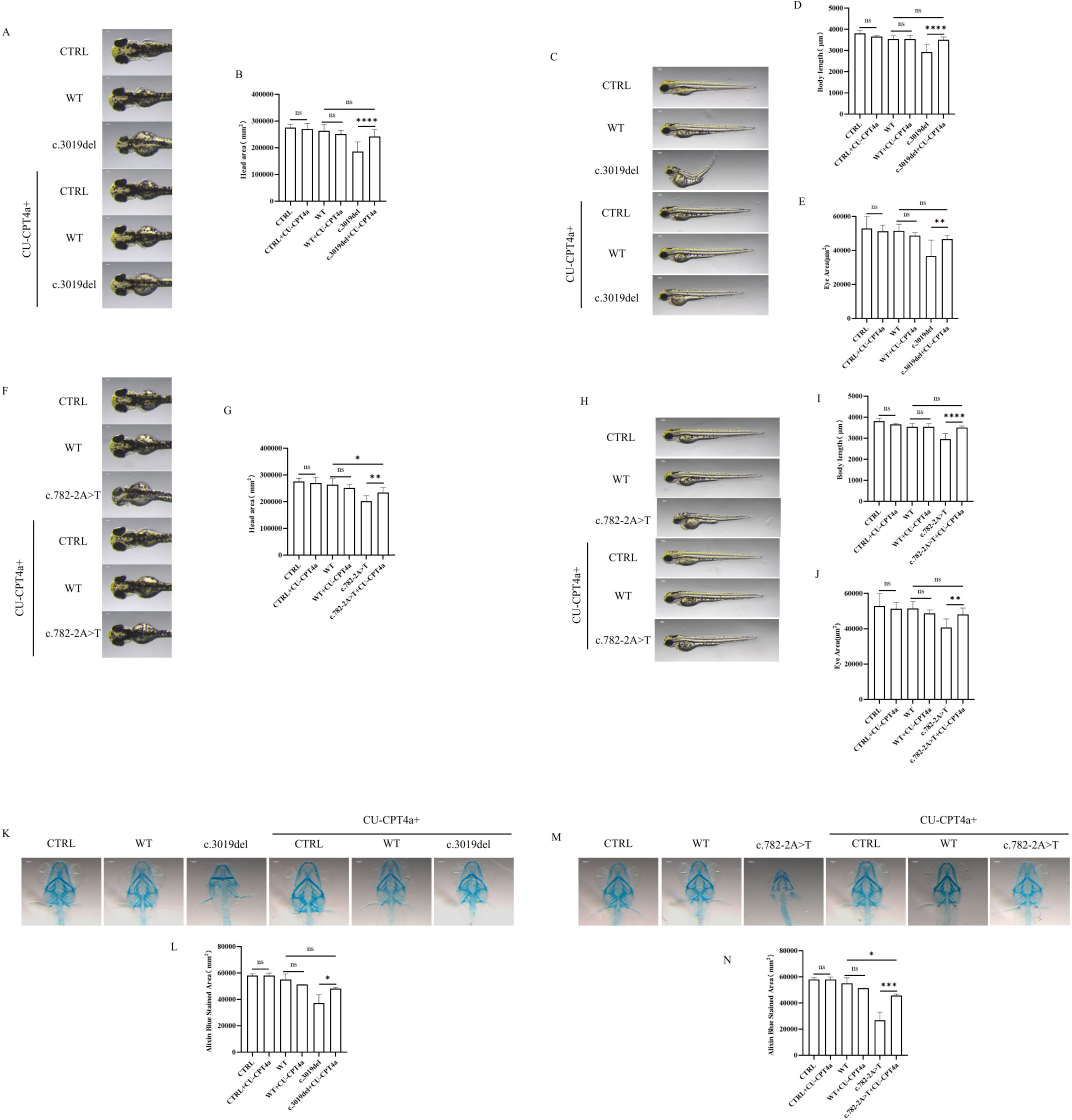
Phenotypic analysis of zebrafish larvae carrying *KDM5C* variants treated with CU-CPT 4a. **(A, F)** Representative head images of CTRL, WT, c.3019del **(A)** or c.782-2A>T **(F)**, and corresponding CU-CPT 4a-treated groups at 72 hpf. **(B, G)** Quantification of head area for CTRL, WT, c.3019del or c.782-2A>T, and their respective CU-CPT 4a-treated groups (*n* =10 per group). **(C, H)** Representative whole-body images of CTRL, WT, c.3019del **(C)** or c.782-2A>T **(H)**, and corresponding CU-CPT 4a-treated groups. **(D, I)** Quantification of body length for the same groups (*n* =10 per group). **(E, J)** Quantification of eye area for the same groups (*n* =10 per group). **(K, M)** Representative alcian blue staining image of CTRL, WT, c.3019del **(K)** or c.782-2A>T **(M)**, corresponding CU-CPT 4a-treated groups at 120 hpf. **(L, N)** Quantification of alcian blue staining area for CTRL, WT, c.3019del or c.782-2A>T, and their respective CU-CPT 4a-treated groups (*n* =3 per group). ANOVA was used to compare data among groups. Data are expressed as mean ± SD. Statistical significance was determined as follows: ns, no significant difference; *P < 0.05; **P < 0.01; ***P < 0.001; ****P < 0.0001.

**Figure 4:**
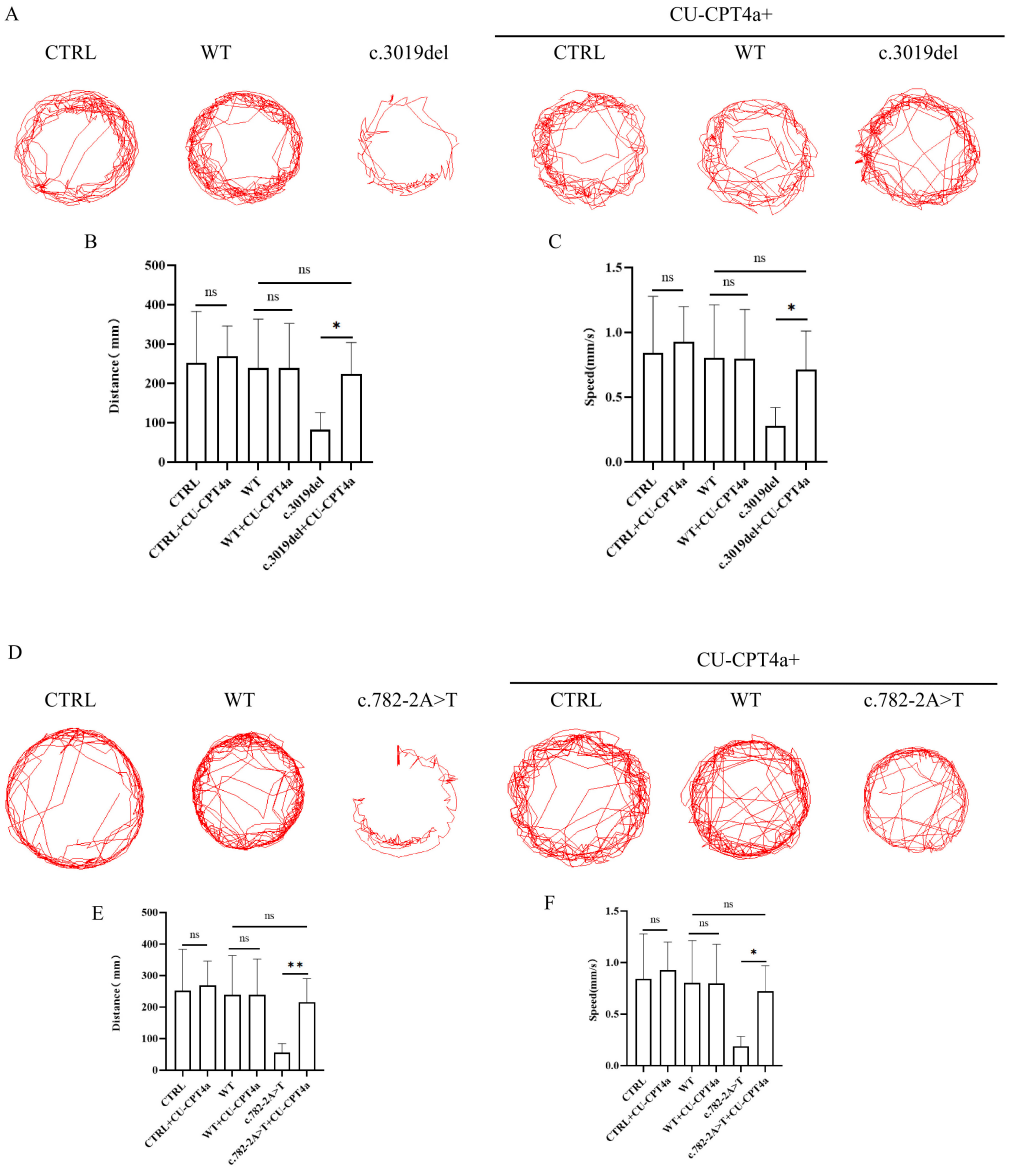
Behavioral assays of zebrafish larvae treated with CU-CPT 4a. **(A, D)** Representative locomotor tracking plots of CTRL, WT, c.3019del **(A)** or c.782-2A>T **(D)**, and their respective CU-CPT 4a-treated groups at 120 hpf. **(B, E)** Quantification of total distance traveled by larvae (*n* = 10 per group) for CTRL, WT, c.3019del or c.782-2A>T, and CU-CPT 4a-treated groups. **(C, F)** Quantification of swimming speed for the same groups. ANOVA was used to compare data among groups. Data are presented as mean ± SD. Statistical significance was determined using appropriate tests: ns, no significant difference; *P < 0.05; **P < 0.01.

To investigate the role of the Toll-like signaling pathway in *KDM5C* mutants, zebrafish larvae carrying c.3019del and c.782-2A>T variants were treated with CU-CPT 4a. Treatment partially restored morphological defects, including head area, body length and eye size in the c.3019del group (Figures 3A–E), with similar improvements observed in the c.782-2A>T group (Figures 3F–J). It is worth noting that after treatment with CU-CPT 4a, there is still a difference in head area between the c.782-2A>T group and the WT group (Figure 3F). Alcian blue staining showed that the cartilage size and structure of the c.3019del variant improved after CU-CPT 4a treatment (Figures 3K, L). Although the c.782-2A>T variant showed improvement after treatment with CU-CPT 4a, there was still a difference compared to the WT group (Figures 3M, N). Behavioral analysis demonstrated that spontaneous swimming activity was restored in both c.3019del and c.782-2A>T zebrafish after CU-CPT 4a treatment (Figure 4), indicating functional rescue. These results suggest that the Toll-like signaling pathway is a key regulator of *KDM5C*-mediated neural development and that inhibition of this pathway can mitigate variant-induced neurodevelopmental abnormalities.

To explore the underlying mechanism, six key Toll-like pathway genes (*TLR3, NFKB1, IFNB1, IRF7, SAT1a*, and *SAT1b*) were analyzed by RT-PCR. mRNA levels of all six genes were upregulated in c.3019del and c.782-2A>T zebrafish compared with WT, and CU-CPT 4a treatment effectively reduced their expression in both c.3019del+CU-CPT 4a and c.782-2A>T+CU-CPT 4a groups (Supplementary Figure 7). Among them, there was no significant difference in the mRNA expression levels of *TLR3* and *NFKB1* between the c.3019del+CU-CPT 4a group and the WT group. The mRNA expression levels of *TLR3* in the c.782-2A>T+CU-CPT 4a group were also not statistically different from those in the WT group. These results suggest that the Toll like receptor signaling pathway, particularly the *TLR3* gene, may be involved in the process of *KDM5C* related functional defects.

## Discussion

In this study, we identified and characterized two novel variants of the *KDM5C* gene in two families with an X-linked ID, combining clinical evaluation, functional assays, and pathway analyses. Pedigree analysis revealed that male hemizygotes in two variant families exhibited short stature, small head circumference, delayed language development and ID, while female carriers presented with learning difficulties and short stature.

Unlike male hemizygotes, the clinical manifestations in heterozygous females are highly variable. More than half of females remain asymptomatic, while others may present with intellectual disability, developmental delay, learning and language difficulties, hormonal disturbances, or anxiety, as reported in recent literature (Lippa et al., 2021; Shen et al., 2022). The basis of this incomplete penetrance in females is not fully understood, although it is widely attributed to the process of XCI (Agulnik et al., 1994). Normally, XCI occurs randomly, with approximately half of a woman's somatic cells silencing either the maternal or paternal X chromosome in roughly half of somatic cells (Amos-Landgraf et al., 2006), with exceptions including escape from inactivation and skewed XCI, the latter being relatively uncommon (Amos-Landgraf et al., 2006). In our study, all female carriers of *KDM5C* variants exhibited skewed XCI in peripheral blood, with preferential expression of the wild-type allele. Despite this, all female carriers still exhibited short stature, and some individuals also exhibited learning difficulties. These results are consistent with prior studies suggesting that XCI status alone does not fully explain phenotypic variability in females with *KDM5C* variants (Carmignac et al., 2020; Shen et al., 2022). One possible explanation is that XCI patterns may differ across tissues, such that peripheral blood may not accurately reflect the situation in brain or other relevant tissues. In addition, genetic modifiers, non-genetic influences, and epigenetic regulation likely contribute to the observed heterogeneity (Carmignac et al., 2020).

Among the variants, c.3019del is a frameshift mutation in the PLU-1 domain, while c.782-2A>T is a splice-site mutation in the linker region between the ARID and PHD1 domains (Supplementary Figure 8). ClinVar lists approximately 236 pathogenic and 82 likely pathogenic *KDM5C* variants, with missense mutations most frequent and splice-site variant rare. Functional characterization of splice-site variants remains limited. Minigene assays of c.782-2A>T revealed aberrant splicing that introduces a premature termination codon in exon 7, predicting a truncated ~375 amino acid protein and disruption of the PHD zinc finger domain essential for *KDM5C* function.

*KDM5C* demethylates H3K4me2/3, regulating transcription at promoters of active or primed genes (Barski et al., 2007; Vallianatos and Iwase, 2015). And loss of this activity has been linked to cognitive impairment (Jensen et al., 2010; Brookes et al., 2015). This activity depends on JmjN and JmjC domains, which are capable of enzymatically removing dimethyl and trimethyl marks from H3K4 (Hatch and Secombe, 2022). In our study, both variants preserved H3K4me3 levels, indicating retained demethylase activity, but significantly reduced *KDM5C* protein stability. These findings suggest that pathogenic effects may occur via non-enzymatic mechanisms (Tahiliani et al., 2007; Vallianatos et al., 2018). Consistent with this, KDM5 family members regulate transcription independently of histone demethylation through interactions with lysine deacetylases and chromatin remodeling complexes (Lee et al., 2009; Mariani et al., 2016). Together, these results highlight *KDM5C*'s dual role in transcriptional regulation and neural development, with variants potentially contributing to cognitive dysfunction through both enzymatic and structural mechanisms.

Neurodevelopmental disorders arise from complex interactions involving genetic, immunological, and inflammatory factors. Maternal immune activation can disrupt fetal neurodevelopment (Szabó et al., 2025) and *KDM5* family involved in in both tumorigenesis and inflammation (Blair et al., 2016; Harmeyer et al., 2017; Liu et al., 2019). *KDM5C* regulates dendritic cell (DC) heterogeneity, influencing the functional characteristics of plasmacytoid (pDC) and classical dendritic cells (cDC) (Boukhaled et al., 2016; Wu et al., 2018; Fazazi et al., 2023), innate immune activity and immune gene expression in non-immune cell. Loss of *KDM5C* in DCs increased inflammatory genes expression, impaired cellular function, and dysregulated lineage-specific gene expression (Guak et al., 2024). *KDM5C* specifically regulates the IRF signaling axis, highlighting its role in immune homeostasis. Loss of function mutations in histone demethylase *KDM5* family is one of the genetic factors leading to intellectual disability and autism spectrum disorder. Neuroinflammation and neuroimmune abnormalities have now been established in autism spectrum disorder as key factors in its development and maintenance (Siniscalco et al., 2018). Research has shown that *Drosophila* deficient in kdm5 display gut dysbiosis, abnormal social behavior, and aberrant immune activation, which Lactobacillus plantarum L168 administration can improve (Chen et al., 2019). When *KDM5* family function is lost, it can disrupt this immune regulatory pathway, leading to dysbiosis of the gut microbiota and chronic neuroinflammation, ultimately promoting the occurrence and development of autism spectrum disorder. Although no significant autistic behavioral characteristics were observed in the two families carrying the *KDM5C* variant in this study, this may be related to the phenotypic diversity of gene variations. The GlinGen working group has previously confirmed that the *KDM5C* gene is a dose sensitive gene, and its pathogenic mechanism is usually manifested as a loss of function type. However, in this study, zebrafish overexpressing *KDM5C* mutants showed significant changes in both phenotype and behavior, while WT *KDM5C* overexpressing zebrafish had the same phenotype as the control group, with no changes in phenotype or behavior. This may be due to the pathogenic mechanism of *KDM5C* mutation with dominant negative effects, but further experiments are needed to verify it.

GO analysis of HEK293 cells expressing c.3019del or c.782-2A>T variants revealed significant upregulation of interferon-mediated signaling and type I interferon receptor binding. KEGG pathway analysis implicated TLR and NOD-like receptor signaling pathways, both critical for interferon responses. TLR signaling involves MyD88, TOLLIP, IL-1 IRAK, and TRAF6 (Medzhitov, 2001), and occurs in immune and neural cells. TLRs contribute to neurogenesis, synaptic plasticity, and cognitive functions (Abarca-Merlin et al., 2024) with aberrant activation during development linked to neurodevelopmental disorders. Neuronal *TLR3* activation inhibits dendritic growth and alters synaptic structure (Zengeler and Lukens, 2021). Based on these findings, we hypothesized that *KDM5C* variants disrupt neurodevelopment by dysregulating Toll-like signaling.

Six genes in the Toll like pathway including *TLR3, IRF7, NFKB1, IFNB1, STAT1*, and *STAT2*, were selected for *in vivo* validation. *TLR3* serves as the initial sensor, *IRF7* amplifies signaling, *NFKB1* and *IRF7* mediate early transcriptional responses, *IFNB1* connects pathogen recognition to antiviral signaling, and *STAT1/STAT2* execute late-stage antiviral effector functions. RNA profiling demonstrated significant upregulation of all six genes in cells expressing pathogenic *KDM5C* variants, confirming activation of innate immune and inflammatory pathways. Notably, pharmacological inhibition of Toll-like signaling using CU-CPT 4a rescued both phenotypic and behavioral abnormalities in zebrafish models carrying these variants, suggesting a potential therapeutic approach.

While immune dysregulation emerges as a key contributor, further studies are needed to explore tissue-specific XCI, validate findings in mammalian models, and develop therapeutic strategies. The partial rescue of mutant phenotypes in zebrafish by Toll-like pathway inhibition highlights neuroinflammation and neuroimmunity as a promising target for intervention in *KDM5C*-associated intellectual disability. Targeting the regulation of TRL related receptors (such as *TLR3*) may become a potential strategy for treating *KDM5C* variants related neurodevelopmental disorders by reducing inflammation and neuroimmunity to improve disease progression.

## Data Availability

The datasets presented in this study can be found in online repositories. The names of the repository/repositories and accession number(s) can be found in the article/Supplementary material.

## Supplementary material

Supplementary Figure 1 XCI analysis in female carriers. Red arrows indicate X chromosomes of maternal origin, and green arrows indicate those of paternal origin. **(A)** XCI analysis in female carriers of Family 1. (i) PCR before digestion in the proband's mother (II-6); (ii) PCR after digestion in the proband's mother (II-6); (iii) PCR before digestion in the proband's aunt (II-2); (iv) PCR after digestion in the proband's aunt (II-2); (v) PCR before digestion in the proband's grandmother (I-2); (vi) PCR after digestion in the proband's grandmother (I-2). **(B)** XCI analysis in female carriers of Family 2. (i) PCR before digestion in the proband's sister (III-3); (ii) PCR after digestion in the proband's sister (III-3); (iii) PCR before digestion in the proband's mother (II-4); (iv) PCR after digestion in the proband's mother (II-4).

Supplementary Figure 2 *In vitro* functional analysis of splicing defects induced by *KDM5C* c.782-2A>T mutations. **(A)** Sanger sequencing of the splicing product from HeLa cells transfected with the mutant construct (HeLa-c.782-2A>T) reveals a 5 bp deletion at the exon 6-exon 7 junction. **(B)** Sanger sequencing of the product from 293T cells transfected with the mutant construct (293-c.782-2A>T) confirms the same 5 bp deletion at the exon 6-exon 7 junction. **(C)** Schematic representation of the minigene construct, RT-PCR primer design and the resulting splicing pattern. The red arrow indicates the mutation site, which potentially disrupts normal splicing.

Supplementary Figure 3 Structural modeling and expression analysis of *KDM5C* variants. **(A)** Predicted three-dimensional structure of the wild-type *KDM5C* protein, serving as the reference model. **(B)** Structural consequences of the c.3019del variant on the *KDM5C* protein, indicating potential alterations in protein folding and function. **(C)** Structural impact of the c.782-2A>T variant on the *KDM5C* protein, highlighting possible impairment of protein stability or molecular interactions. **(D, E)** *KDM5C* RNA expression in HEK293 cells transfected with control (CTRL), empty vector (NC), wild-type (WT), c.782-2A>T and c.3019del plasmids. **(F)** *KDM5C* protein expression in HEK293 cells transfected with control, empty vector, wild-type, c.3019del and c.782-2A>T plasmids. ANOVA was used to compare data among groups. Data are presented as mean ± SD. ns, no significant difference; ^****^*p* < 0.0001. Relative expression levels were calculated by the 2^−ΔΔ^Ct method and normalized to GAPDH. Data are presented as fold change relative to the control group.

Supplementary Figure 4 Functional effects of *KDM5C* variants on histone methylation and protein stability. Whole-cell lysates from HEK293 cells transfected with control (CTRL), wild-type (WT), c.3019del, or c.782-2A>T constructs were analyzed for H3K4me3 and H3K4me2 expression. (B) Quantification of H3K4me2 levels showing that c.3019del and c.782-2A>T variants did not significantly alter H3K4me2 expression. (C) Quantification of H3K4me3 levels showing that c.3019del and c.782-2A>T variants did not significantly alter H3K4me3 expression. (D) Cycloheximide (CHX) chase assay performed to assess protein stability. (E) Quantification of relative *KDM5C* protein levels following CHX treatment (N = 3). ANOVA was used to compare data among groups. Data are presented as mean ± SD. ns, no significant difference. CHX, cycloheximide; H3K4, histone H3 lysine 4; WT, wild type. Mean gray values of H3K4me2/H3K4me3/KDM5C and GAPDH were measured in ImageJ software and the ratio (H3K4me2/H3K4me3/KDM5C)/GAPDH was calculated and expressed as fold change to control.

Supplementary Figure 5 Analysis of histone H3K4 methylation in zebrafish larvae. **(A)** Western blot analysis of whole-cell lysates from CTRL, wild-type WT, c.3019del, and c.782-2A>T zebrafish showing expression levels of H3K4me3 and H3K4me2. **(B)** Quantification of H3K4me2 levels, demonstrating that c.3019del and c.782-2A>T did not significantly alter H3K4me2 expression. **(C)** Quantification of H3K4me3 levels, showing that c.3019del and c.782-2A>T did not significantly affect H3K4me3 expression. ANOVA was used to compare data among groups. Data are presented as mean ± SD. ns, no significant difference. Mean gray values of H3K4me2/H3K4me3 and GAPDH were measured in ImageJ software and the ratio (H3K4me2/H3K4me3)/GAPDH was calculated and expressed as fold change to control.

Supplementary Figure 6 Transcriptomic analysis of *KDM5C*-c.3019del and *KDM5C*-c.782-2A>T in HEK293 cells. **(A)** Heatmap of microarray data comparing gene expression between c.3019del and controls. Expression levels of 363 genes are shown, with red and blue indicating high and low expression, respectively. **(B**) Volcano plot of gene expression in c.3019del, with red, blue, and gray points representing upregulated, downregulated, and unchanged genes, respectively. **(C)** Gene Ontology (GO) functional enrichment analysis of differentially expressed genes in c.3019del versus controls. **(D)** KEGG pathway analysis showing the top 27 pathways enriched among differentially expressed genes in c.3019del. **(E)** Heatmap of microarray data comparing gene expression between c.782-2A>T and controls. Expression levels of 326 genes are shown, with red and blue indicating high and low expression, respectively. **(F)** Volcano plot of gene expression in c.782-2A>T, with red, blue, and gray points representing upregulated, downregulated, and unchanged genes, respectively**. (G)** GO functional enrichment analysis of differentially expressed genes in c.782-2A>T versus controls. **(H)** KEGG pathway analysis showing the top 27 pathways enriched among differentially expressed genes in c.782-2A>T.

Supplementary Figure 7 Effects of CU-CPT 4a on mRNA expression of immune and inflammation genes in zebrafish larvae. **(A)** Relative mRNA expression levels of *TLR3, NFKB1, IFNB1, IRF7, SAT1a*, and *SAT1b* in CTRL, CTRL+CU-CPT 4a, WT, WT+CU-CPT 4a, c.3019del, and c.3019del+CU-CPT 4a groups. **(B)** Relative mRNA expression levels of *TLR3, NFKB1, IFNB1, IRF7, SAT1a*, and *SAT1b* in CTRL, CTRL+CU-CPT 4a, WT, WT+CU-CPT 4a, c.782-2A>T, and c.782-2A>T+CU-CPT 4a groups. ANOVA was used to compare data among groups. Data are presented as mean ± SD. Statistical significance was determined using appropriate tests: ns, no significant difference; ^*^*P* < 0.05; ^**^*P* < 0.01; ^***^*P* < 0.001; ^****^*P* < 0.0001.

Supplementary Figure 8 Schematic representation of the *KDM5C* protein based on uniprot web (https://www.uniprot.org/). The positions indicated by the black solid arrows correspond to the locations of two novel variants, while the positions indicated by the dashed arrows correspond to the locations of amino acid changes resulting from the c.782-2A>T variant. JmjN, N-terminal Jumonji domain; ARID, AT-rich interaction domain; PHD, PHD Zinc finger; JmjC, C-terminal Jumonji domain; ZF, Zinc Finger domain; PLU-1, PLU-1-like protein.
