# Supplementary figures and images for: Emerging role of KDM5C in X-linked intellectual disability based on human genetic data and zebrafish models
Source: Front Mol Neurosci. 2026 Feb 10;19:1750311. doi: 10.3389/fnmol.2026.1750311 (PMC12929123; doi:10.3389/fnmol.2026.1750311)

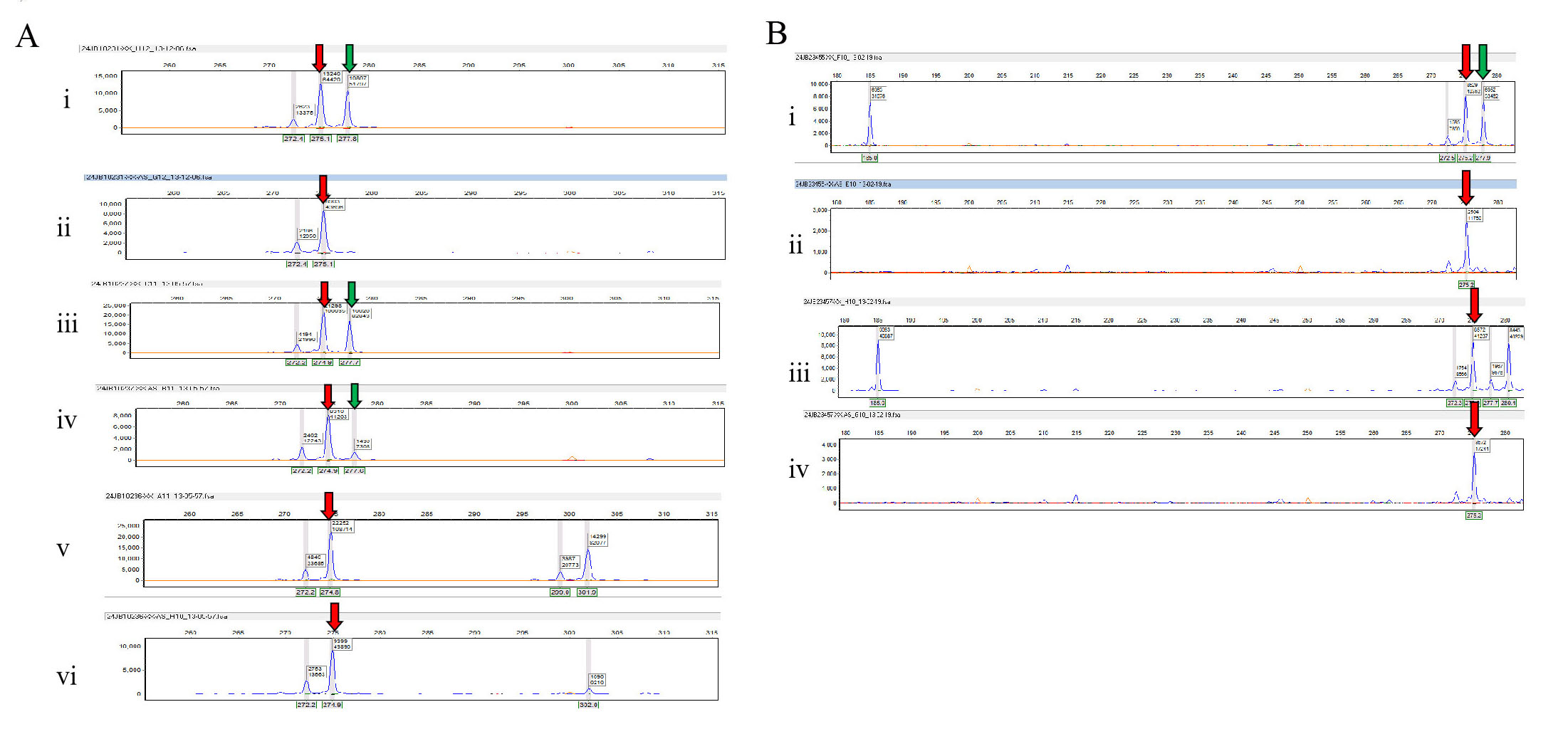

Supplement: Supplementary Figure 1 — XCI analysis in female carriers. Red arrows indicate X chromosomes of maternal origin, and green arrows indicate those of paternal origin. (A) XCI analysis in female carriers of Family 1. (i) PCR before digestion in the proband's mother (II-6); (ii) PCR after digestion in the proband's mother (II-6); (iii) PCR before digestion in the proband's aunt (II-2); (iv) PCR after digestion in the proband's aunt (II-2); (v) PCR before digestion in the proband's grandmother (I-2); (vi) PCR after digestion in the proband's grandmother (I-2). (B) XCI analysis in female carriers of Family 2. (i) PCR before digestion in the proband's sister (III-3); (ii) PCR after digestion in the proband's sister (III-3); (iii) PCR before digestion in the proband's mother (II-4); (iv) PCR after digestion in the proband's mother (II-4). [file Image_1.jpeg]

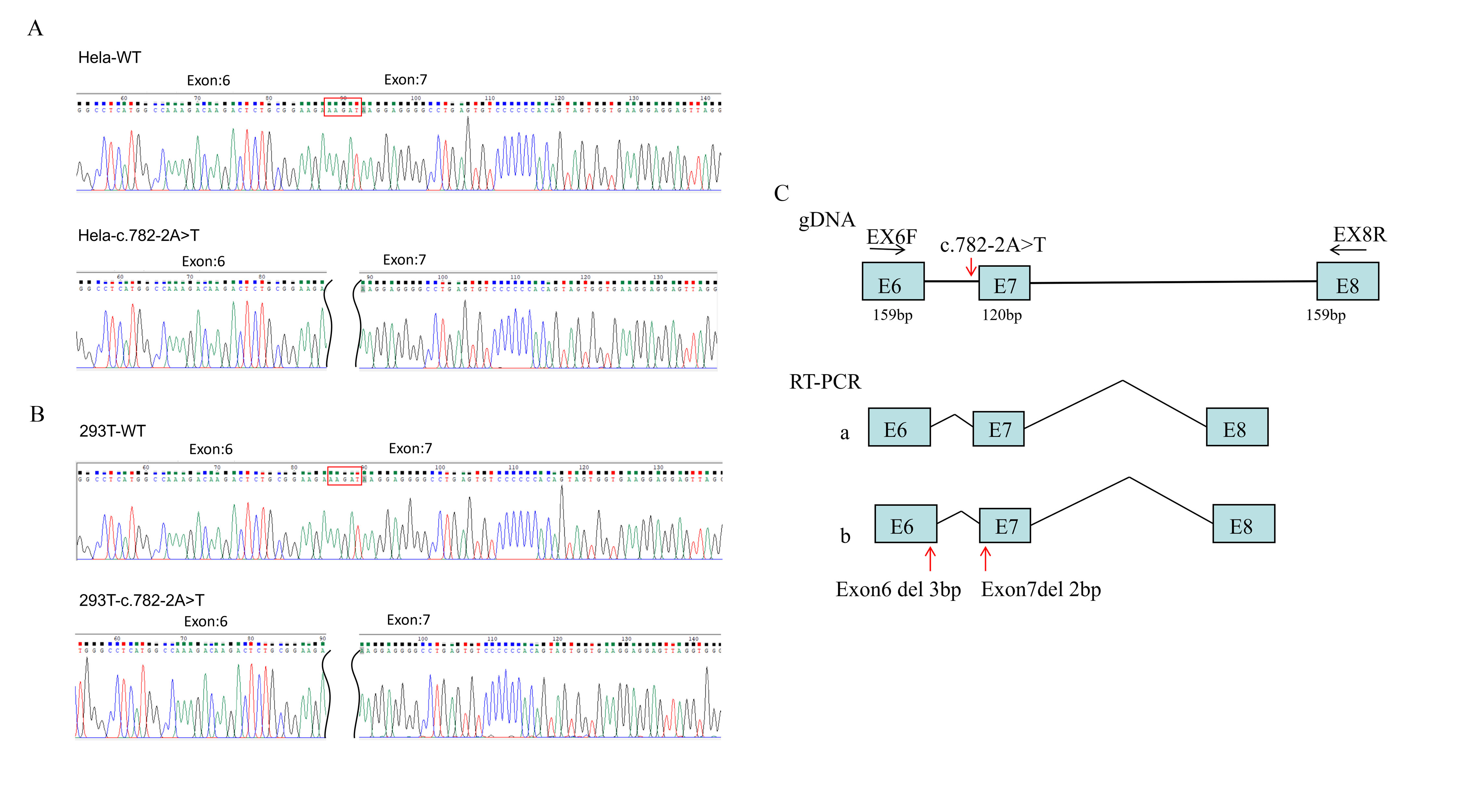

Supplement: Supplementary Figure 2 — In vitro functional analysis of splicing defects induced by KDM5C c.782-2A>T mutations. (A) Sanger sequencing of the splicing product from HeLa cells transfected with the mutant construct (HeLa-c.782-2A>T) reveals a 5 bp deletion at the exon 6-exon 7 junction. (B) Sanger sequencing of the product from 293T cells transfected with the mutant construct (293-c.782-2A>T) confirms the same 5 bp deletion at the exon 6-exon 7 junction. (C) Schematic representation of the minigene construct, RT-PCR primer design and the resulting splicing pattern. The red arrow indicates the mutation site, which potentially disrupts normal splicing. [file Image_2.jpg]

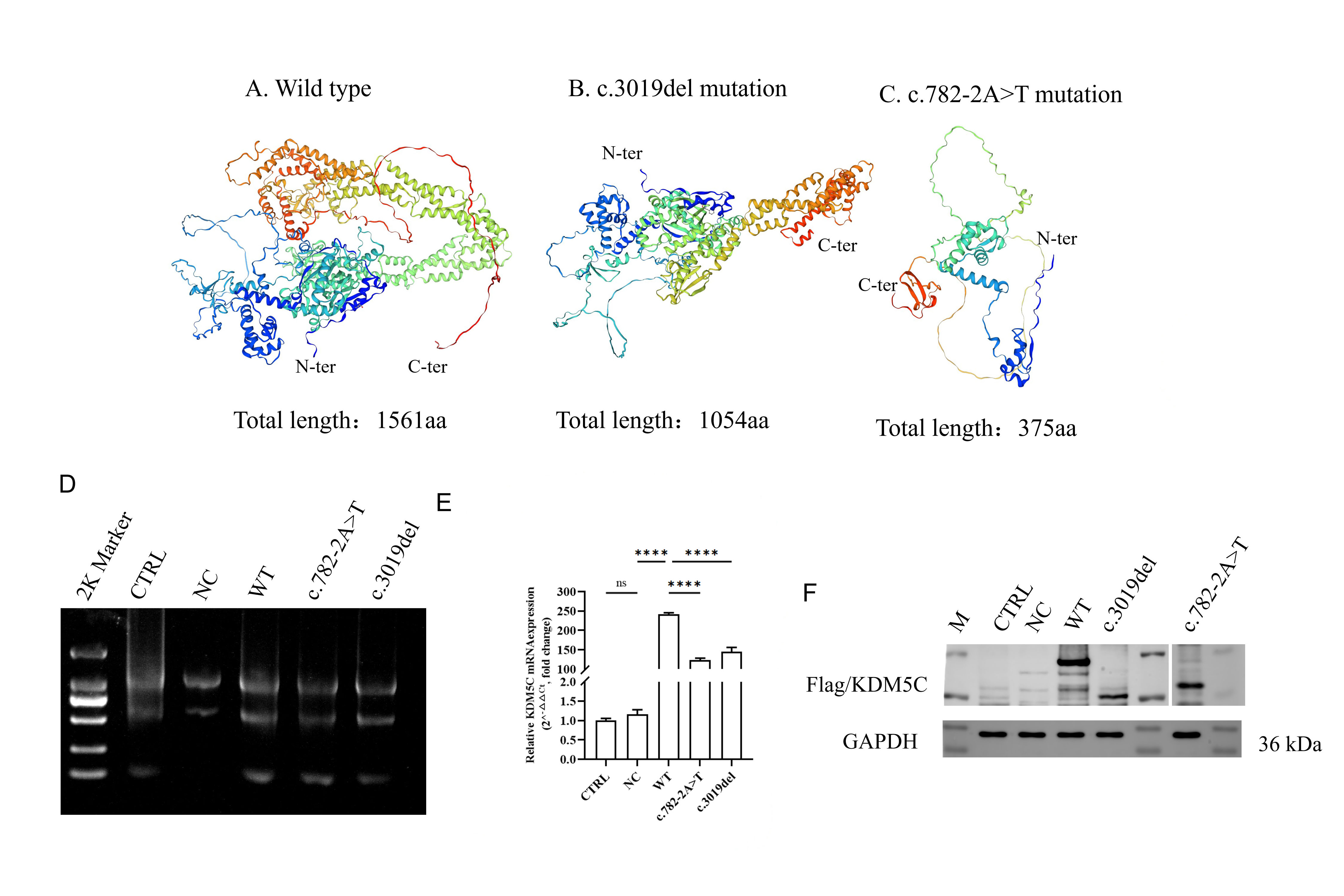

Supplement: Supplementary Figure 3 — Structural modeling and expression analysis of KDM5C variants. (A) Predicted three-dimensional structure of the wild-type KDM5C protein, serving as the reference model. (B) Structural consequences of the c.3019del variant on the KDM5C protein, indicating potential alterations in protein folding and function. (C) Structural impact of the c.782-2A>T variant on the KDM5C protein, highlighting possible impairment of protein stability or molecular interactions. (D, E) KDM5C RNA expression in HEK293 cells transfected with control (CTRL), empty vector (NC), wild-type (WT), c.782-2A>T and c.3019del plasmids. (F) KDM5C protein expression in HEK293 cells transfected with control, empty vector, wild-type, c.3019del and c.782-2A>T plasmids. ANOVA was used to compare data among groups. Data are presented as mean ± SD. ns, no significant difference; ****p < 0.0001. Relative expression levels were calculated by the 2−ΔΔCt method and normalized to GAPDH. Data are presented as fold change relative to the control group. [file Image_3.jpg]

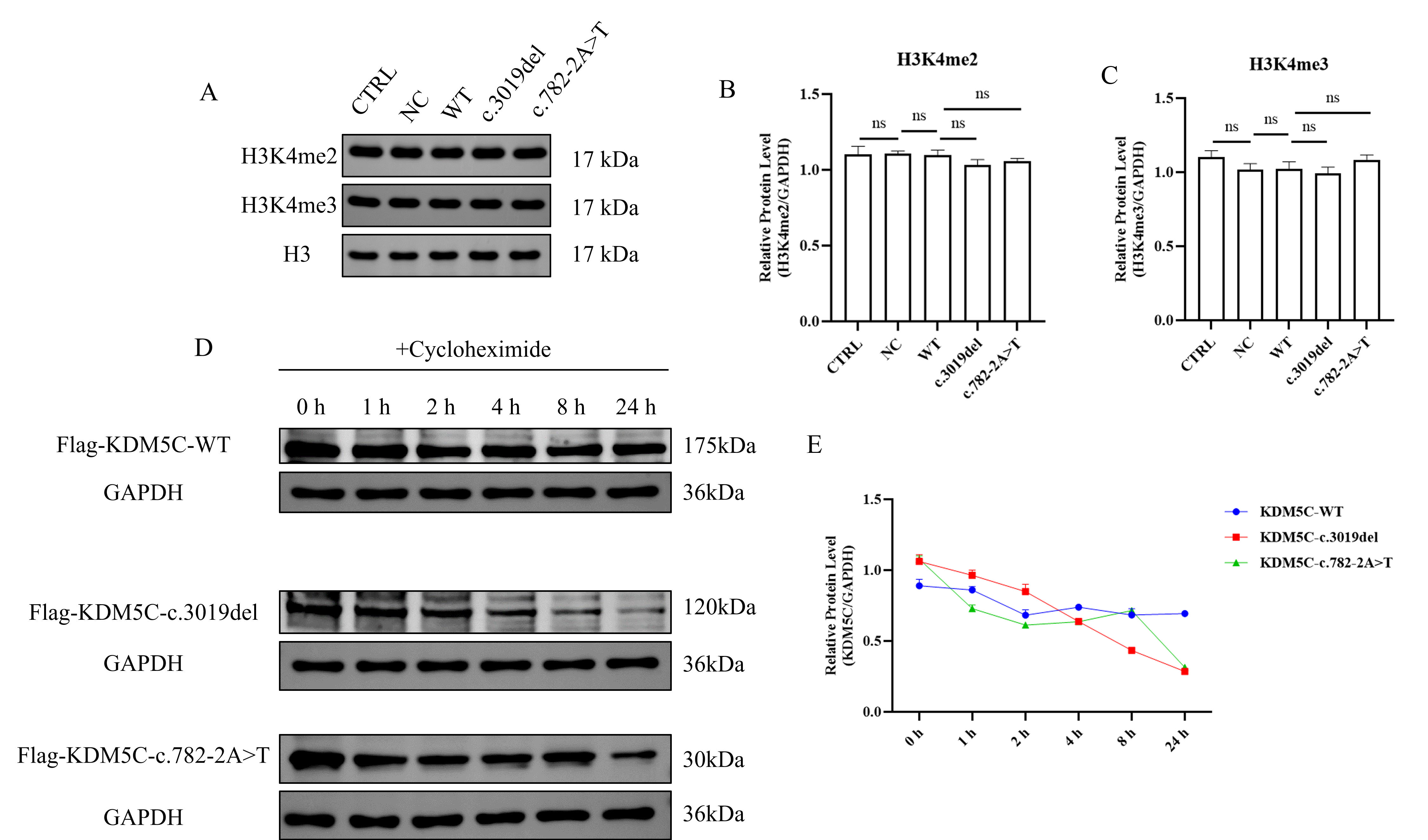

Supplement: Supplementary Figure 4 — Functional effects of KDM5C variants on histone methylation and protein stability. Whole-cell lysates from HEK293 cells transfected with control (CTRL), wild-type (WT), c.3019del, or c.782-2A>T constructs were analyzed for H3K4me3 and H3K4me2 expression. (B) Quantification of H3K4me2 levels showing that c.3019del and c.782-2A>T variants did not significantly alter H3K4me2 expression. (C) Quantification of H3K4me3 levels showing that c.3019del and c.782-2A>T variants did not significantly alter H3K4me3 expression. (D) Cycloheximide (CHX) chase assay performed to assess protein stability. (E) Quantification of relative KDM5C protein levels following CHX treatment (N = 3). ANOVA was used to compare data among groups. Data are presented as mean ± SD. ns, no significant difference. CHX, cycloheximide; H3K4, histone H3 lysine 4; WT, wild type. Mean gray values of H3K4me2/H3K4me3/KDM5C and GAPDH were measured in ImageJ software and the ratio (H3K4me2/H3K4me3/KDM5C)/GAPDH was calculated and expressed as fold change to control. [file Image_4.jpg]

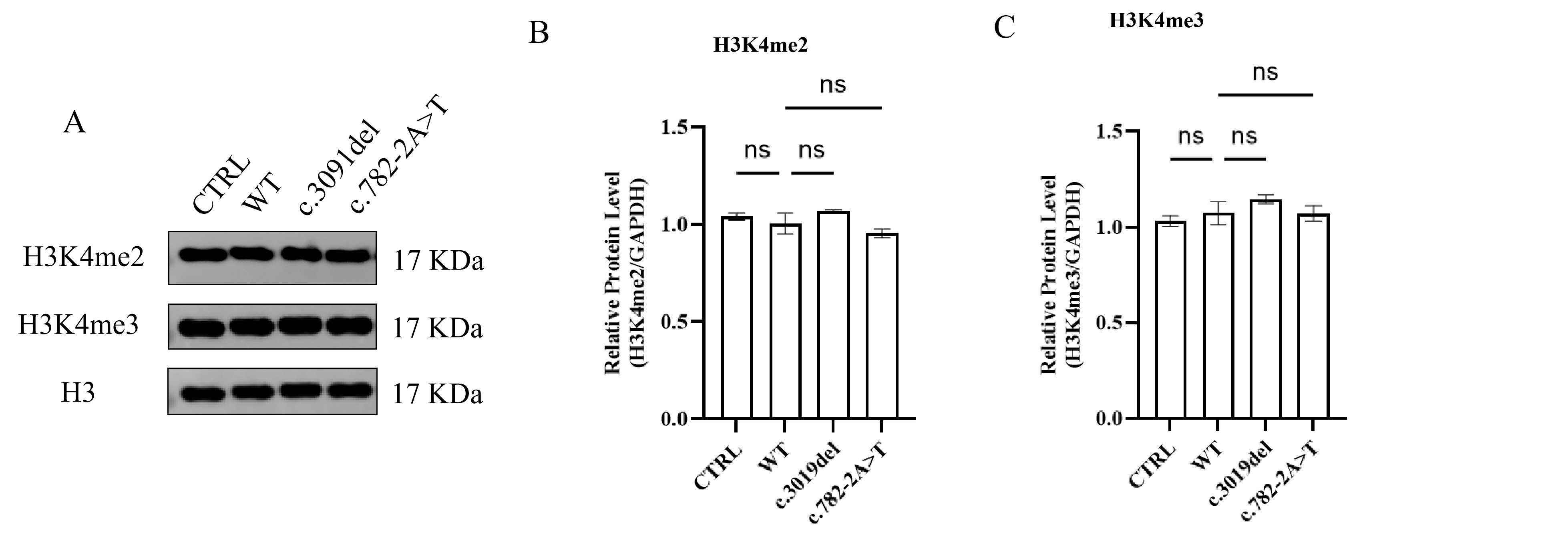

Supplement: Supplementary Figure 5 — Analysis of histone H3K4 methylation in zebrafish larvae. (A) Western blot analysis of whole-cell lysates from CTRL, wild-type WT, c.3019del, and c.782-2A>T zebrafish showing expression levels of H3K4me3 and H3K4me2. (B) Quantification of H3K4me2 levels, demonstrating that c.3019del and c.782-2A>T did not significantly alter H3K4me2 expression. (C) Quantification of H3K4me3 levels, showing that c.3019del and c.782-2A>T did not significantly affect H3K4me3 expression. ANOVA was used to compare data among groups. Data are presented as mean ± SD. ns, no significant difference. Mean gray values of H3K4me2/H3K4me3 and GAPDH were measured in ImageJ software and the ratio (H3K4me2/H3K4me3)/GAPDH was calculated and expressed as fold change to control. [file Image_5.jpg]

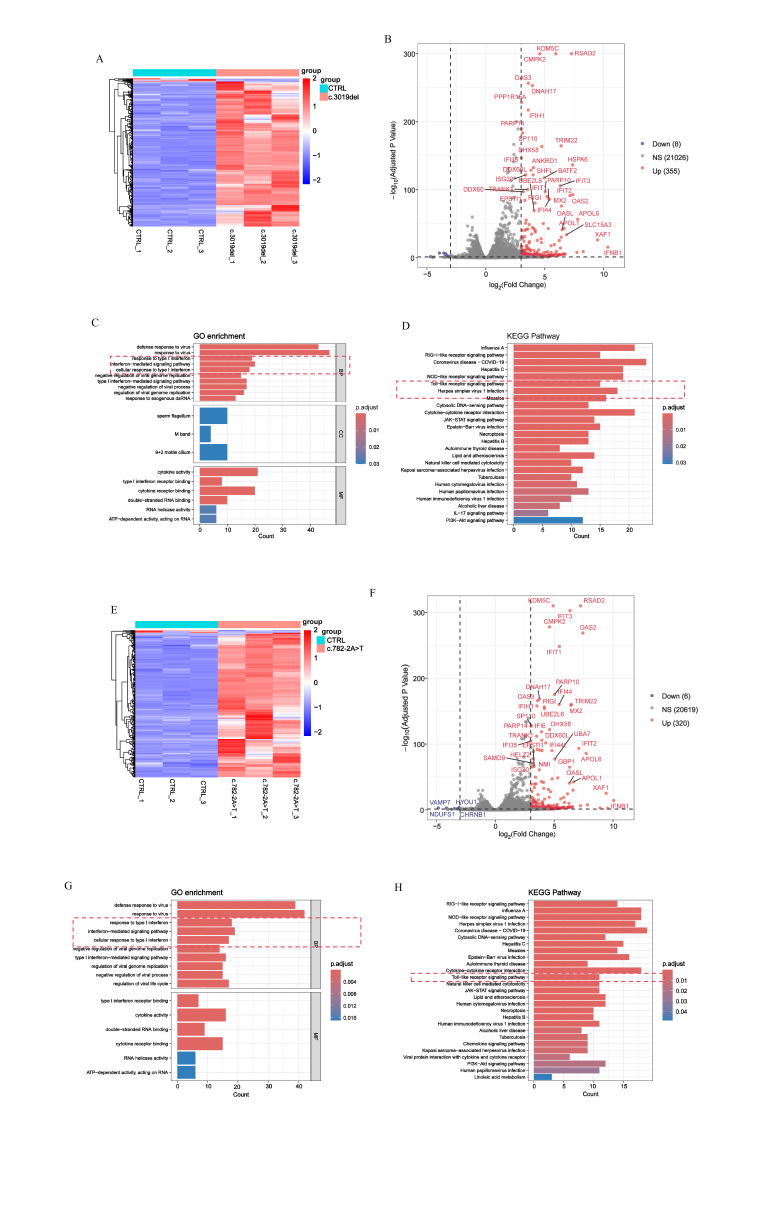

Supplement: Supplementary Figure 6 — Transcriptomic analysis of KDM5C-c.3019del and KDM5C-c.782-2A>T in HEK293 cells. (A) Heatmap of microarray data comparing gene expression between c.3019del and controls. Expression levels of 363 genes are shown, with red and blue indicating high and low expression, respectively. (B) Volcano plot of gene expression in c.3019del, with red, blue, and gray points representing upregulated, downregulated, and unchanged genes, respectively. (C) Gene Ontology (GO) functional enrichment analysis of differentially expressed genes in c.3019del versus controls. (D) KEGG pathway analysis showing the top 27 pathways enriched among differentially expressed genes in c.3019del. (E) Heatmap of microarray data comparing gene expression between c.782-2A>T and controls. Expression levels of 326 genes are shown, with red and blue indicating high and low expression, respectively. (F) Volcano plot of gene expression in c.782-2A>T, with red, blue, and gray points representing upregulated, downregulated, and unchanged genes, respectively. (G) GO functional enrichment analysis of differentially expressed genes in c.782-2A>T versus controls. (H) KEGG pathway analysis showing the top 27 pathways enriched among differentially expressed genes in c.782-2A>T. [file Image_6.jpg]

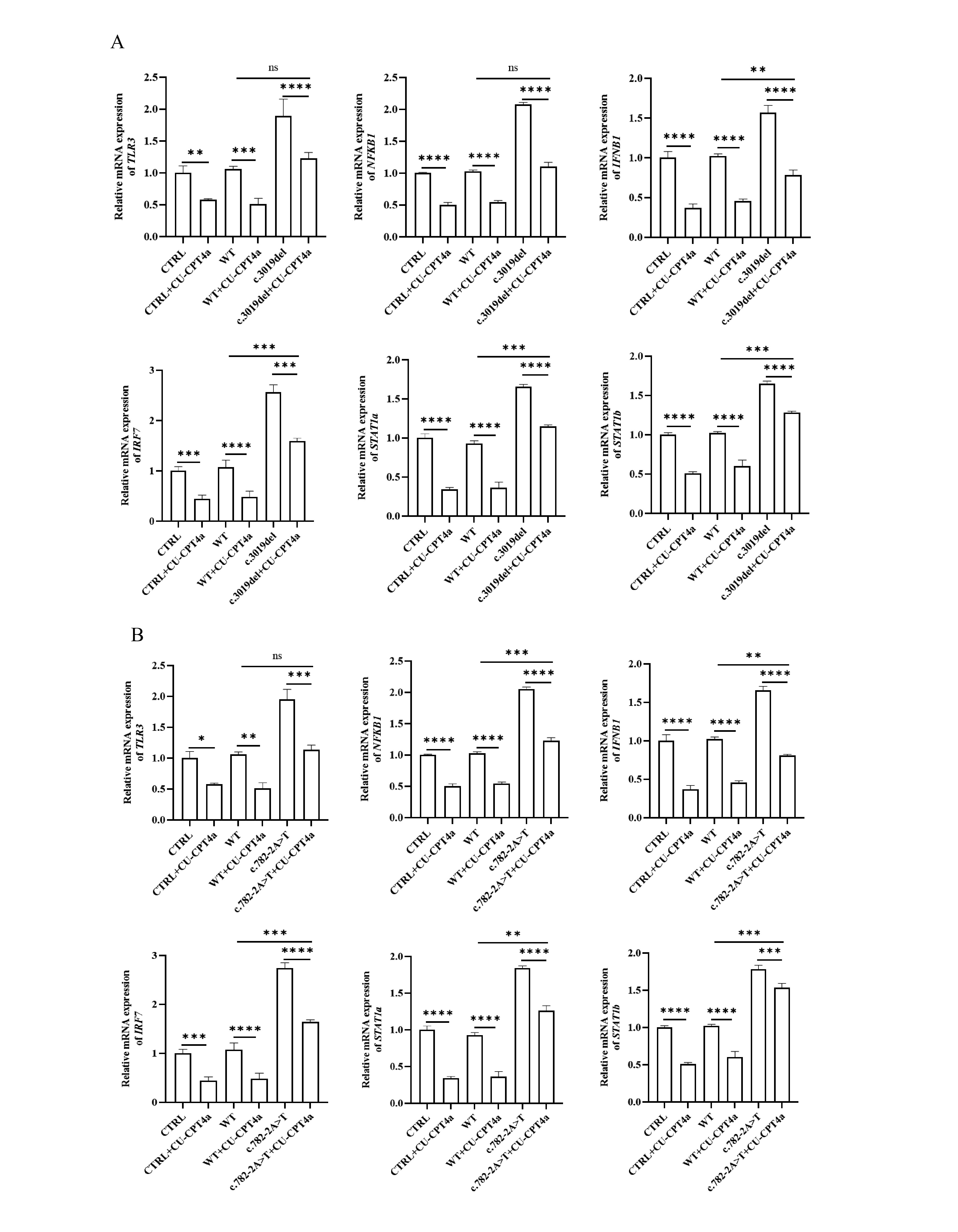

Supplement: Supplementary Figure 7 — Effects of CU-CPT 4a on mRNA expression of immune and inflammation genes in zebrafish larvae. (A) Relative mRNA expression levels of TLR3, NFKB1, IFNB1, IRF7, SAT1a, and SAT1b in CTRL, CTRL+CU-CPT 4a, WT, WT+CU-CPT 4a, c.3019del, and c.3019del+CU-CPT 4a groups. (B) Relative mRNA expression levels of TLR3, NFKB1, IFNB1, IRF7, SAT1a, and SAT1b in CTRL, CTRL+CU-CPT 4a, WT, WT+CU-CPT 4a, c.782-2A>T, and c.782-2A>T+CU-CPT 4a groups. ANOVA was used to compare data among groups. Data are presented as mean ± SD. Statistical significance was determined using appropriate tests: ns, no significant difference; *P < 0.05; **P < 0.01; ***P < 0.001; ****P < 0.0001. [file Image_7.jpeg]

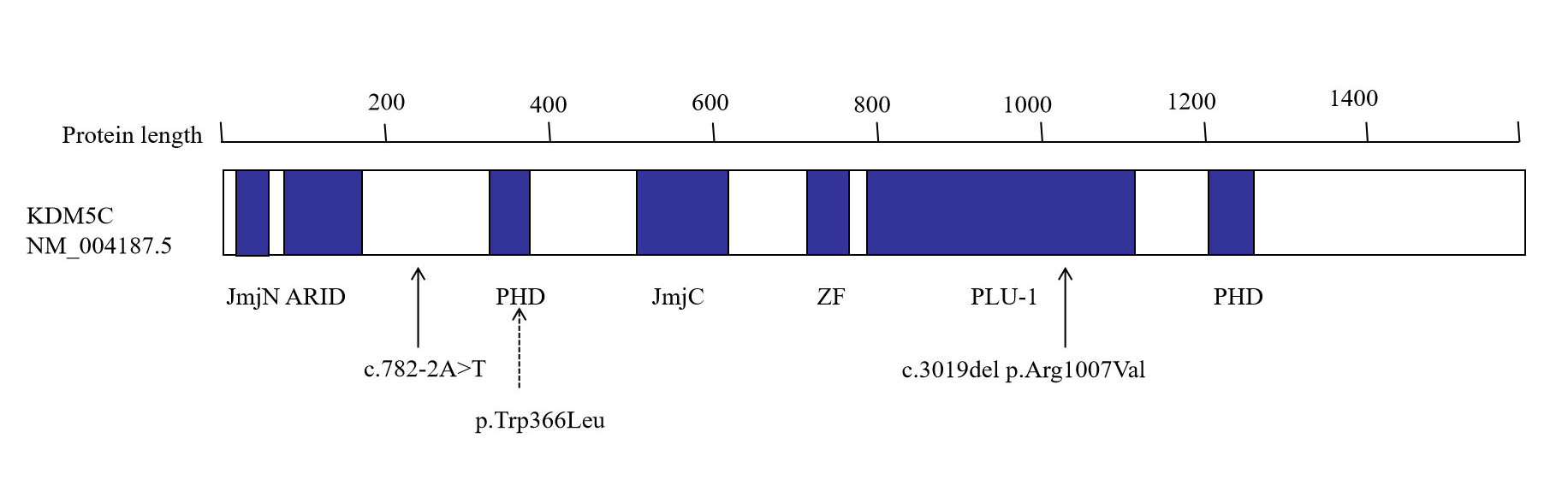

Supplement: Supplementary Figure 8 — Schematic representation of the KDM5C protein based on uniprot web (https://www.uniprot.org/). The positions indicated by the black solid arrows correspond to the locations of two novel variants, while the positions indicated by the dashed arrows correspond to the locations of amino acid changes resulting from the c.782-2A>T variant. JmjN, N-terminal Jumonji domain; ARID, AT-rich interaction domain; PHD, PHD Zinc finger; JmjC, C-terminal Jumonji domain; ZF, Zinc Finger domain; PLU-1, PLU-1-like protein. [file Image_8.jpeg]
