# Supplementary material for: Emerging role of KDM5C in X-linked intellectual disability based on human genetic data and zebrafish models
Source: Front Mol Neurosci. 2026 Feb 10;19:1750311. doi: 10.3389/fnmol.2026.1750311 (PMC12929123; doi:10.3389/fnmol.2026.1750311)
Supplement: Supplementary file 9 [file Table_1.doc]

**eTable 1** Primer sequences for RT-PCR analysis of over-expression in cells experiment

| Primers | Primer sequence（5’-3’） |
| --- | --- |
| H-*GAPDH*-F | TCAAGAAGGTGGTGAAGCAGG |
| H-*GAPDH*-R | TCAAAGGTGGAGGAGTGGGT |
| H-*KDM5C*-F | TGTCCACTTCCACAGCAAAG |
| H-*KDM5C*-R | CTTGGCTACATCGCGAAAAT |

**eTable 2 Exploring the optimal concentration for overexpression**

| embryos | 6hpf | 24hpf | | 48hpf | |
| --- | --- | --- | --- | --- | --- |
| Group-concentration (ng/μL) | Mortality rate | Mortality rate | Malformation rate | Mortality rate | Malformation rate |
| WT-100 | 0.87% | 3.04% | 0.00% | 3.91% | 1.81% |
| WT-300 | 4.25% | 7.34% | 0.00% | 8.88% | 2.12% |
| WT-600 | 5.42% | 8.33% | 0.45% | 10.42% | 5.58% |
| c.3019del-100 | 4.68% | 14.47% | 1.99% | 17.02% | 10.26% |
| c.3019del-300 | 7.54% | 21.43% | 3.54% | 24.60% | 13.16% |
| c.3019del-600 | 11.00% | 55.00% | 10.00% | 60.00% | 33.75% |
| c.782-2A>T-100 | 8.51% | 20.21% | 14.00% | 22.34% | 15.07% |
| c.782-2A>T-300 | 9.42% | 25.56% | 13.25% | 28.70% | 20.75% |
| c.782-2A>T-600 | 18.05% | 56.10% | 24.44% | 64.88% | 45.83% |

**eTable 3** Primer sequences for RT-PCR analysis of over-expression and rescue experiment

| Primers | Primer sequence（5’-3’） |
| --- | --- |
| *β-actin*-F | GTATTGCTGACCGTATGC |
| *β-actin*-R | CTGCCTCATCGTATTCCT |
| *H-KDM5C*-F | TGTCCACTTCCACAGCAAAG |
| *H-KDM5C*-R | CTTGGCTACATCGCGAAAAT |

**eTable 4 Primer sequences for RT-PCR analysis of CU-CPT 4a treatment experiment**

| Primers | Primer sequence（5’-3’） |
| --- | --- |
| *zTLR3*-F | CTGACACAGCCTTGCGAAAC |
| *zTLR3*-R | AATTCCAGTTGGGGCAACCA |
| *zNFKB1*-F | CTTCACGCTCACAGTCTGGT |
| *zNFKB1*-R | GATGCTGGAGTCTTTGGGCT |
| *zIFNB1*-F | AAGGTGAATCTTGAGGAAAGTGAG |
| *zIFNB1*-R | TTGAACATGTGCCAGCCTCT |
| *zIRF7*-F | ACATACCTGCTTCAGTCCAGC |
| *zIRF7*-R | GGAAGCGTATTTGCTCCCCT |
| *zSTAT1a*-F | TGTCCGAGGTGTTGAACCTG |
| *zSTAT1a*-R | AGCCATCTGTTGACGACGTT |
| *zSTAT1b*-F | ACCACCTAATGCGTGTCTGG |
| *zSTAT1b*-R | CTGCCTCTCCACCACAAGAG |
| *zβactin-F* | GTATTGCTGACCGTATGC |
| *zβactin-R* | CTGCCTCATCGTATTCCT |
